# Finding optimum climatic parameters for high tomato yield in Benin (West Africa) using frequent pattern growth algorithm

**DOI:** 10.1371/journal.pone.0297983

**Published:** 2024-02-08

**Authors:** Sèton Calmette Ariane Houetohossou, Vinasetan Ratheil Houndji, Rachidatou Sikirou, Romain Glèlè Kakaï

**Affiliations:** 1 Laboratoire de Biomathématiques et d’Estimations Forestières, University of Abomey-Calavi, Cotonou, Benin; 2 Institut de Formation et de Recherche en Informatique, University of Abomey-Calavi, Cotonou, Benin; 3 Laboratoire de Défense des Cultures, Centre de Recherches Agricoles d’Agonkanmey, Institut National des Recherches Agricoles du Bénin (INRAB), Cotonou, Republic of Benin; University 20 Aout 1955 skikda, Algeria, ALGERIA

## Abstract

Tomato is one of the most appreciated vegetables in the world. Predicting its yield and optimizing its culture is important for global food security. This paper addresses the challenge of finding optimum climatic values for a high tomato yield. The Frequent Pattern Growth (FPG) algorithm was considered to establish the associations between six climate variables: minimum and maximum temperatures, maximum humidity, sunshine (*Sun*), rainfall, and evapotranspiration (*ET*), collected over 26 years in the three agro-ecological Zones of Benin. Monthly climate data were aggregated with yield data over the same period. After aggregation, the data were transformed into ‘low’, ‘medium’, and ‘high’ attributes using the threshold values defined. Then, the rules were generated using the minimum support set to 0.2 and the confidence to 0.8. Only the rules with the consequence ‘high yield’ were screened. The best yield patterns were observed in the Guinean Zone, followed by the Sudanian. The results indicated that high tomato yield was associated with low *ET* in all areas considered. Minimum and maximum temperatures, maximum humidity, and *Sun* were medium in every Zone. Moreover, rainfall was high in the Sudanian Zone, unlike the other regions where it remained medium. These results are useful in assessing climate variability’s impact on tomato production. Thus, they can help farmers make informed decisions on cultivation practices to optimize production in a changing environment. In addition, the findings of this study can be considered in other regions and adapted to other crops.

## Introduction

Agriculture is a significant component of the economy of developing countries. It contributes 30 to 60 percent of the gross domestic product (GDP) in about two-thirds of these countries [1]. In Benin, it is the most important sector, providing up to 36% of the GDP [2]. In particular, vegetable production is essential in reducing poverty by increasing employment opportunities and promoting the country’s economic development [3]. The top five vegetables for investments in Benin are tomato (*Solanum lycopersicum* L.), chilli pepper (*Capsicum frutescens* L.), habanero pepper (*Capsicum chinense* J.), onion (*Allium cepa* L.), and carrot (*Daucus carota* L.) [4]. Among them, tomatoes are the most important regarding area and production [5]. It is very appreciated because of its usefulness for most cooked foods [4].

Farmers need to predict yield in advance to be more efficient and maximize profits. Predicting yield is essential for agricultural risk management and future forecasting decisions. *Yield* depends on the climate, soil, water, nutrient availability, diseases and pests management, farming practices, choice of variety, and growing methods [6]. Statistical methods such as simple and multiple linear regressions can be used to predict crop yield. Bahrami [7] using Backward Multiple Linear Regressions based on the Relative Importance Metrics to determine the effect of climatic parameters on the rainfall yield of wheat showed that the duration of sunshine was the most important parameter for growth. Linear regression models assume a linear relationship between the independent and dependent variables. However, the relationships between the predictors and dependent variables can be non-linear and complex. Machine learning models, on the other hand, overcome this shortcoming [8]. Various machine learning approaches have been actively employed in recent studies related to yield prediction, owing to the non-linear spatiotemporal nature of crop yields [9]. Machine Learning is a component of Artificial Intelligence that has proven effective in providing concrete solutions to many challenges in agriculture, including yield prediction, disease detection, weed detection, crop quality, and species recognition [10]. For example, [11] applied machine learning models to predict the yield of six crops: rice, maize, cassava, cotton, yams, and bananas in some West African countries. For this purpose, they used historical data on the yield of the target crops as well as climate, weather, and chemical data. The Decision Tree model performed best with a coefficient of determination of 95.3%. In addition, Gómez [12] used satellite and climate data to model wheat yield in Mexico. After testing several ML models, the Random Forest (RF) provided better prediction. Moreover, using a LASSO, RF, XGBoost regression method and the Long Short Term Memory (LSTM), Zhang [13] developed an approach that integrated optical, fluorescence, satellite thermal, and environmental data to predict maize yield in four agro-ecological Zones in China and showed that combining these multi-source data explained more than 75% of the yield variation. Furthermore, Rao [14] used three association algorithms, Apriori, Eclat, and AprioriTid, to determine the climatic and soil attributes that contribute to the excellent performance of paddy rice in India. They found that the Eclat algorithm performed better than the other two.

Also, in India, a prediction model was proposed to forecast crop yield in the districts of Tamil Nadu based on available previous data [15]. The Apriori algorithm was applied to a pre-processed dataset obtained through the modified K-means algorithm to predict the yield of various crops. Moreover, Supro [16] used the Apriori algorithm to generate association rules on the attributes area, production, yield, temperature, precipitation, humidity, and wind speed for predicting paddy crop yield in the Larkana district of India.

The aforementioned studies have focused on cereals. No work links climate attributes to tomato yield in Africa, although this could help optimize production. Indeed, by using weather data, farmers can predict tomato yield and adjust their agricultural practices accordingly. This can help maximize production by adapting the amount of water and fertilizers applied and predicting market fluctuations. In addition, predicting tomato yields based on weather conditions can help farmers better manage the risks associated with extreme weather conditions, such as droughts, floods, or winds. Accurate forecasts can help farmers protect their crops, reduce production losses, and minimize economic impacts. Moreover, farmers can plan yields and forecast stocks to ensure a constant and stable market supply, thus contributing to food security [17].

In this paper, we propose to use an association rule algorithm, frequent pattern growth (Fp growth), to find the optimum climatic attributes to maximize tomato yield in Benin. In data mining, association rule mining is a popular and extensively studied method for discovering relationships of interest between two or more variables stored in large databases [18]. The study used weather and tomato yield data collected in Benin’s three agroecological zones over a period of 26 years. The weather data considered are temperature, humidity, sunshine, and rainfall.

The remaining sections of the paper are organized in the following manner. The first section briefly outlines the literature’s most prevalent association rules approaches. The second section describes the proposed methodology, highlighting the study area’s presentation and the database used, the pre-processing of this data, and the metric used to evaluate the chosen approach. The third and fourth sessions present and discuss the main results obtained. Finally, the last session concludes.

## Brief description of the most common association rule algorithms

Association rules use Machine Learning models to analyze datasets for patterns or co-occurrences in a database. An association rule has two components: an antecedent (if) and a consequent (then), and it identifies frequent ‘if-then’ associations, which are association rules. The rules are computed from itemsets consisting of two or more elements. Popular algorithms that use association rules include Apriori, Equivalence Class Clustering bottom-up Lattice Traversal (ECLAT), and Frequent Pattern Growth (Fp Growth). A brief description of the three algorithms is provided below.

### Apriori

Created by Agrawal [19], the Apriori algorithm is a multi-pass database form whose initial practical application is to recommend products based on ones already in the user’s cart. It enables finding frequent patterns among the elements stored in a database to generate association rules from these common elements. Its fundamental principle is that all non-empty subsets of a set of frequent elements must also be frequent. To do this, it starts by finding candidate item sets and combining each item with every other item in the preceding item set. The frequent item set is then generated from the previous candidate item set by pruning the items whose support does not meet the selected minimum threshold. Finally, it uses a bottom-up approach known as candidate sets, where frequent sets are extended individually. The main disadvantage is that it requires several database scans to calculate each item’s support.

### ECLAT

The ECLAT algorithm works vertically, like a deep graph search, making it faster than the Apriori algorithm. The primary purpose is to use the intersections of Transaction Id Sets(tidsets) to calculate a candidate’s support value and avoid the generation of subsets that are not present in the prefix tree [20]. When the function is at the first call, all unique elements are used with their tidsets. After that, the procedure is called recursively, and in each recursive call, each element-tidset pair is checked and combined with other element-tidset couples. The process is repeated until no candidate item-tidset pair can be combined.

### Fp growth

The Frequent Patterns Growth algorithm finds sets of frequent items without generating candidates. It works in two steps. The first step consists of constructing a compact data structure called FP-Tree. The second step is devoted to directly extracting frequent sets from the FP-Tree. The FP-Tree has been proposed by Kamber [21]. Each path in the FP-Tree represents a set of relevant frequent information, and the paths’ nodes are arranged in descending order of their frequency. In the FP-tree, the information in the dataset is highly compressed because all overlapping itemsets share the same prefix path [22]. It traverses the database only twice and does not require candidate generation.

## Materials and methods

This section explains the study environment and the data used. It illustrates the variation of these data in time, presents the preprocessing, and details the technique and performance metrics.

### Study area and dataset presentation

**Fig 1:**
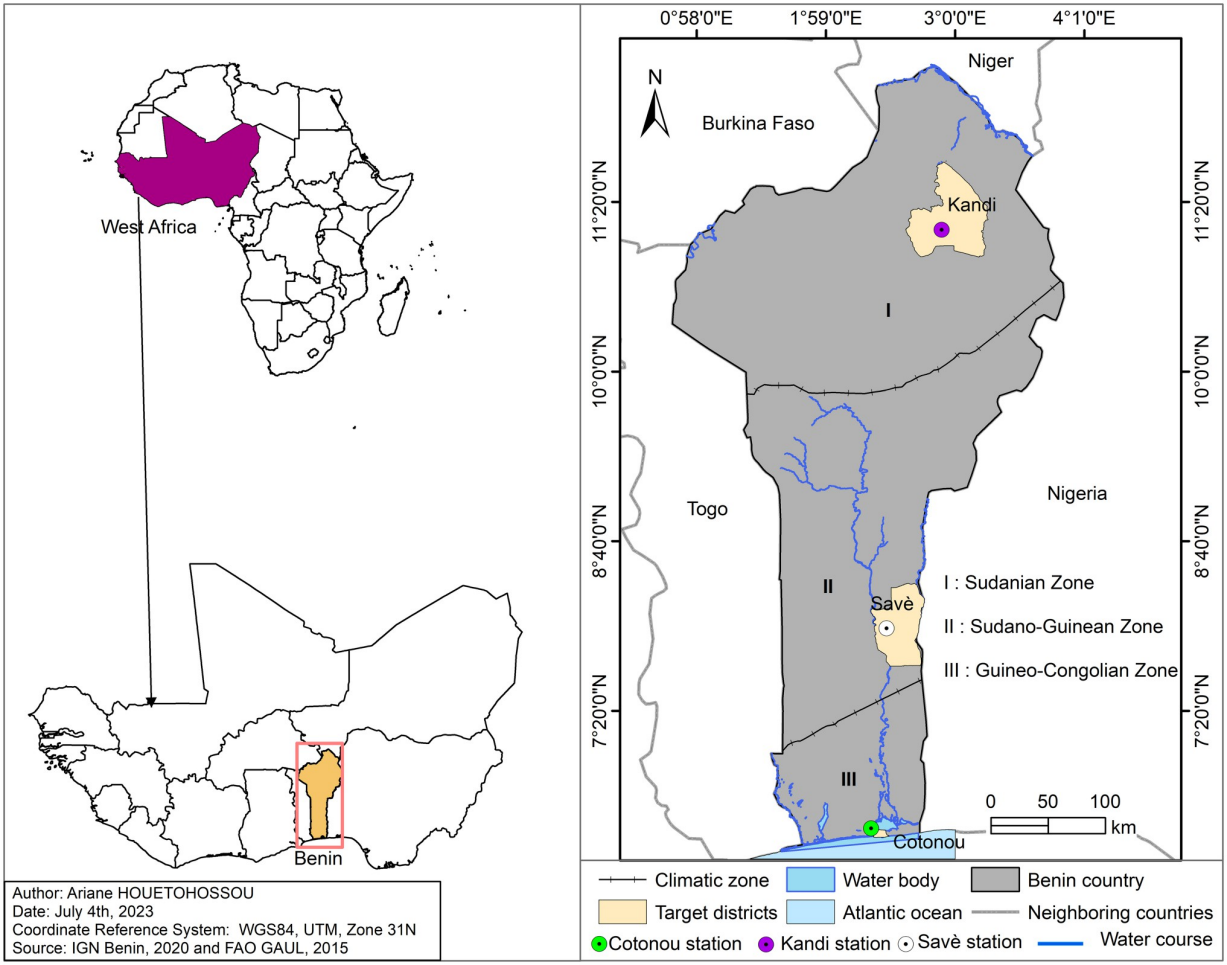
Map of Africa and Benin Republic.

The country of study is the Republic of Benin, a West African country (Fig 1). We used secondary data collected at the Kandi, Savè, and Cotonou synoptic stations in the Sudanian, Sudano-Guinean, and Guinean Zones, respectively. The government of Benin has defined two poles for vegetable production: poles 1 and 7. Pole 1 is located in the Sudanian Zone and covers the districts of Malanville and Karimama, which do not have synoptic stations. Therefore, we chose the synoptic station of Kandi, the closest station to Karimama and Malanville and located in the same department as these two districts. Pole 7, located in the Sudanian Zone, covers several communities with a single synoptic station: Cotonou. In addition, data from the synoptic station of Savè, situated in the Sudano-Guinean transition Zone, was collected. The choice of the station of Savè allows the study to cover the three types of climate in the country.

**Fig 2:**
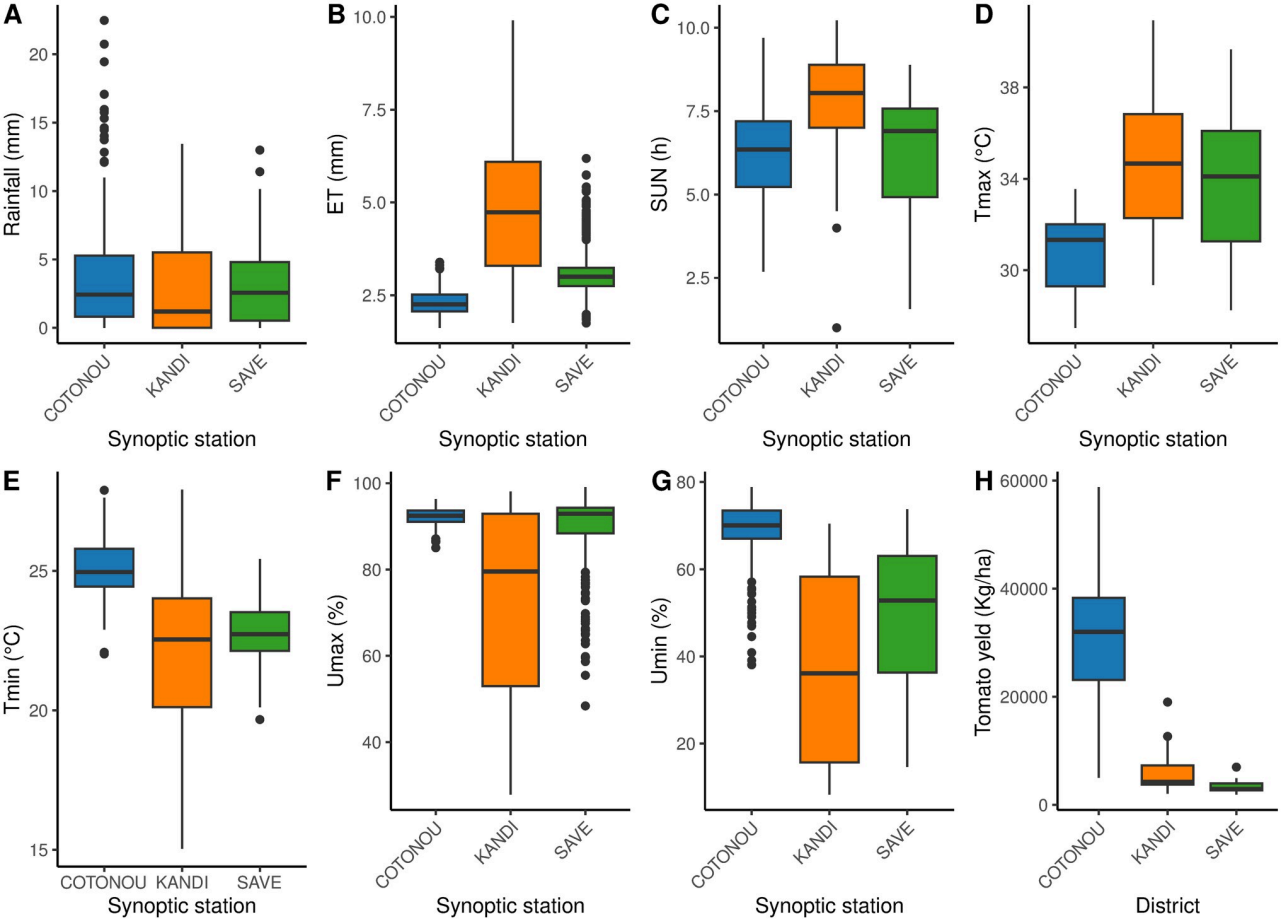
Distribution of climate parameters and tomato yield from 1995 to 2020 in the three districts. **A**: Rainfall. **B**: Evapotranspiration. **C**: Sunstroke. **D**: Maximum temperature. **E**: Minimum temperature. **F**: Maximum humidity. **G**: Minimum humidity. **H**: Tomato yield.

The secondary climate data from the three synoptic stations (Kandi, Savè, and Cotonou) were collected at the ‘Direction de la Météo-Bénin’ of the ‘Agence pour la Sécurité de la Navigation Aérienne en Afrique (ASECNA)’ from 1995 to 2020. These are minimum temperature (*Tmin*) in °C, maximum temperature (*Tmax*) in °C, minimum humidity (*Umin*) in %, maximum humidity (*Umax*) in %, rainfall (*RR*) in mm, sunshine (*Sun*) in hours (h), and evapotranspiration (*ET*) in mm. Annual tomato yield data from 1995 to 2020 was obtained from the ‘Direction de la Statistique Agricole du Benin (DSA)’. The average monthly yield was computed from the annual yields. This monthly yield was aggregated to the monthly average of each climatic data. The final base consists of 936 observations on eight variables, including the yield. Fig 2 shows the data distribution for each parameter and the yield according to the districts. The rainfall boxplots show a similar pattern for the three synoptic stations, with more outliers observed at the Cotonou station (Fig 2A). Sunstroke is higher at the synoptic station of Kandi than at the other two stations (Fig 2C). The last trend is also observed for Evapotranspiration and maximum temperature (Fig 2B & 2D). On the other hand, the minimum temperature, maximum and minimum humidities are more important at the Cotonou station than the other two stations (Fig 2E–2G). Similarly, tomato yields are much higher in Cotonou (Fig 2H).

### Temporal variation of the climatic parameters over the years

**Fig 3:**
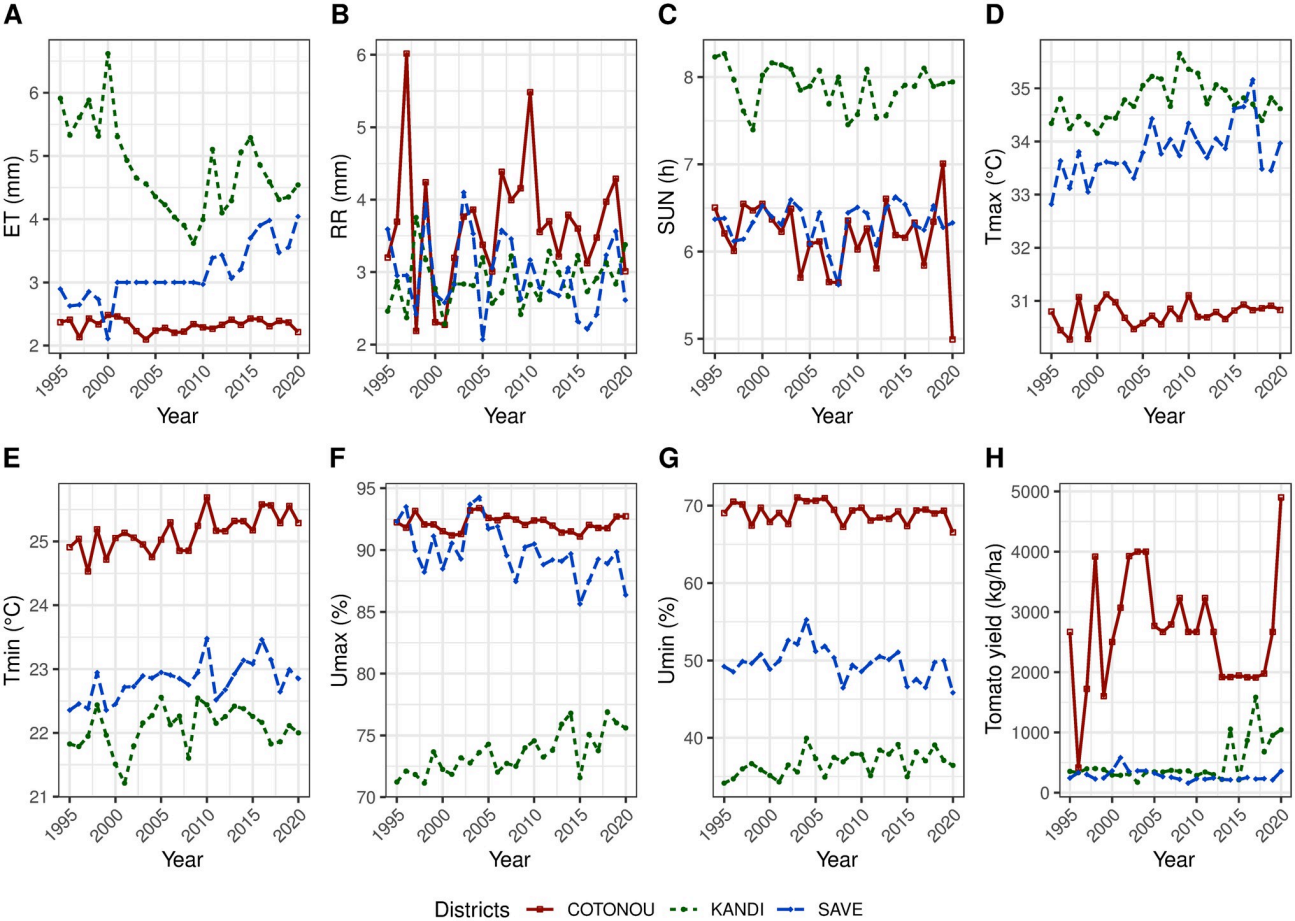
Temporal variation of climate parameters and tomato yield in the three districts. **A**: Rainfall. **B**: Evapotranspiration. **C**: Sunstroke. **D**: Maximum temperature. **E**: Minimum temperature. **F**: Maximum humidity. **G**: Minimum humidity. **H**: Tomato yield.

Fig 3 showed the annual average of the parameters in the study areas over 26 years. Kandi station recorded the highest values of *ET* (6.6 mm), *Tmax* (35.6°C), and *Sun* (8.2 h). The *Sun* trends are similar in Cotonou and Savè. A slight variation was observed in *Tmax* at Cotonou between 1995 and 2015, where *Tmin* and *Umin* were high. However, *Umin* was low in Savè and Kandi. Cotonou and Savè showed the same variations for the *Umax*. The yield was higher up to 5000 kg/ha at Cotonou, while in the other two Zones, it remained at almost 500 kg/ha.

### Data pre-processing

**Fig 4:**
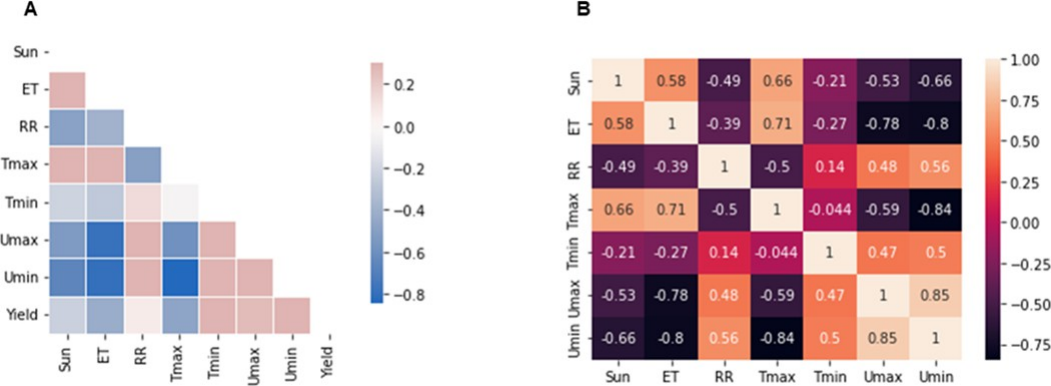
Correlation analysis between variables. **A**: Correlation between predictors and response variable. **B**: Correlation between predictors.

The correlation analysis between the dependent and independent variables was performed using a threshold of 80%. Thus, all variables that correlate above this threshold were removed. No explanatory variable was correlated with the response variable (Fig 4A). However, the correlation analysis between the explanatory variables indicated that the maximum temperature was correlated with the minimum humidity at more than 80%. In addition, there was a high correlation of 85% between maximum and minimum humidity (Fig 4B). Therefore, we removed the minimum humidity from the predictors.

From the daily climate data, we calculated the monthly average of each parameter to have twelve entries per year. This mean was aggregated to the monthly averages of the yield data. This resulted in a matrix of 936 observations on seven attributes, including minimum temperature, maximum temperature, maximum humidity, rainfall, sunshine, evapotranspiration, and tomato yield. The attributes were then categorized as ‘low’, ‘medium’, or ‘high’ based on the respective threshold value specified by the agro-ecological Zone in Tables 1–3. The thresholds were defined by calculating the means ± standard deviation of the data collected over the 26 years by agro-ecological Zone. Tables 1–3 provide information on the threshold values for the Sudanian, Sudano-Guinean, and Guinean Zones for each variable. After this, the attributes were transformed into dummy variables with the package *pandas* available in the free Python software.

**Table 1:** Threshold values of variables for Sudanian Zone.

Attributes			
	Low	Medium	High
*Sun* (h)	≤ 6.727	]6.727; 9.157[	≥ 9.157
*ET* (mm)	≤ 3.009	]3.009; 6.585[	≥ 6.585
*RR* (mm)	≤ 0.109	]0.109; 0.323[	≥ 0.323
*Tmax* (°C)	≤ 31.733	]31.733; 37.793[	≥ 37.793
*Tmin* (°C)	≤ 19.024	]19.024; 25.120[	≥ 25.120
*Umax* (%)	≤ 52.850	]52.850; 94.264[	≥ 94.264
*Yield* (kg/ha)	≤ 192.671	]192.671; 689.989[	≥ 689.989

**Table 2:** Threshold values of variables for Sudano-Guinean Zone.

Attributes			
	Low	Medium	High
*Sun* (h)	≤ 4.664	]4.664; 7.994[	≥ 7.994
*ET* (mm)	≤ 2.387	]2.387; 3.887[	≥ 3.887
*RR* (mm)	≤ 4.504	]4.504; 8.347[	≥ 8.347
*Tmax* (°C)	≤ 31.079	]31.079; 36.545[	≥ 36.545
*Tmin* (°C)	≤ 21.968	]21.968; 23.825[	≥ 23.825
*Umax* (%)	≤ 88.161	]88.161; 95.710[	≥ 95.710
*Yield* (kg/ha)	≤ 208.159	]208.159; 322.760[	≥ 322.760

**Table 3:** Threshold values of variables for the Guinean Zone.

Attributes			
	Low	Medium	High
*Sun* (h)	≤ 4.664	]4.664; 7.528[	≥ 7.528
*ET* (mm)	≤ 1.971	]1.971; 2.673[	≥ 2.673
*RR* (mm)	≤ 1.331	]1.331; 3.250[	≥ 3.250
*Tmax* (°C)	≤ 29.214	]29.214; 32.282[	≥ 32.282
*Tmin* (°C)	≤ 24.424	]24.424; 26.213[	≥ 26.213
*Umax* (%)	≤ 90.036	]90.036; 93.962[	≥ 93.962
*Yield* (kg/ha)	≤ 1739.144	]1739.144; 3618.280[	≥ 3618.280

### The association rule technique used and evaluation metrics

Several algorithms can be used to establish the rules, including Apriori and frequent pattern growth (Fp growth). We focused on FP Growth to establish the association rules because it is more efficient and scalable than the Apriori algorithm [23], and Garg [24] demonstrated that Eclat is less efficient than FP Growth. The minimum support was set to 0.2. We chose a minimum support value that is not too small to avoid the size of frequent itemsets being too large at the expense of execution efficiency [25]. In addition, this value is manageable to prevent insignificant itemsets from being generated [25]. Filtering was performed on rules that contained at least three antecedents and only high yield as a consequent. The rules retained were the five most relevant ones whose confidence is at least 0.8, and lift is greater or equal to 1. Three metrics were used to evaluate the constructed rules: support, confidence, and lift. For the association rule *X* ⇒ *Y*, the support indicates how frequently items *X* and *Y* appear together in the database. The confidence represents the number of times the ‘if-then’ statements are considered trustworthy [26]. The lift measures the importance of a rule by comparing the confidence of the rule with the expected confidence [26]. If the lift is negative, the data points have a negative correlation. If it is at least 1, it indicates a positive correlation between X and Y and the significance of the association. The following formulas were considered to calculate the parameters [26].

$$\begin{array}{c} support\left(X,Y\right)=\frac{Frequency(X,Y)}{N} \end{array}$$ (1)

$$\begin{array}{c} confidence\left(X,Y\right)=\frac{Frequency(X,Y)}{Frequency(X)} \end{array}$$ (2)

$$\begin{array}{c} lift\left(X,Y\right)=\frac{support(X,Y)}{support(X)\times support(Y)} \end{array}$$ (3)

where *X* is the antecedent, *Y* is the consequent and *N* is the total number of transactions.

## Results

The most relevant rules in each agro-ecological Zone define associations between the attributes minimum temperature, maximum temperature, *ET*, sunshine, maximum humidity, and rainfall for high yield of tomato in Benin (Tables 4–6). The rules have been filtered to get out the most pertinent with a consequent: ‘high yield’. The supports for the rules were above the minimum threshold. In addition, each rule established has a confidence of 1 and a lift equal to 1, attesting to their reliability. Overall trends indicated high tomato yield with medium values of minimum temperature, maximum temperature, maximum humidity, and sunshine, whatever the area considered (Tables 4–6). At the same time, *ET* was low (Tables 4–6) and rainfall were high in the Sudanian Zone (Table 4) and medium in the other two Zones (Tables 5 and 6). In the following subsections, we detail the rules separately for each Zone.

**Table 4:** Rules from Sudanian Zone.

Antecedents	Consequent	Support	Confidence	Lift
*ET* Low *Tmin* Medium *Umax* High	*Yield* High	0.250	1.0	1.0
*Tmax* Medium *Umax* Medium *RR* High	*Yield* High	0.208	1.0	1.0
*ET* Low *Umax* Medium *RR* Medium	*Yield* High	0.250	1.0	1.0
*Tmax* Medium *ET* Low *RR* Medium	*Yield* High	0.250	1.0	1.0
*Tmax* Medium *Tmin* Medium *RR* Medium	*Yield* High	0.208	1.0	1.0

**Table 5:** Rules from Sudano–Guinean Zone.

Antecedents	Consequent	Support	Confidence	Lift
*ET* Low *Tmin* Medium *RR* Medium	*Yield* High	0.416	1.0	1.0
*ET* Low *Tmin* Medium *Umax* Medium	*Yield* High	0.309	1.0	1.0
*Tmax* Medium *Tmin* Medium *Umax* Medium	*Yield* High	0.297	1.0	1.0
*Tmin* Medium *Umax* Medium *Sun* Medium	*Yield* High	0.261	1.0	1.0

**Table 6:** Rules from Guinean Zone.

Antecedents	Consequent	Support	Confidence	Lift
*ET* Low *Tmin* Medium *Sun* Medium	*Yield* High	0.457	1.0	1.0
*Tmax* Medium *Tmin* Medium *RR* Medium	*Yield* High	0.237	1.0	1.0
*ET* Low *Umax* Medium *Sun* Medium	*Yield* High	0.423	1.0	1.0
*Tmin* Medium *Umax* Medium *Sun* Medium	*Yield* High	0.288	1.0	1.0
*ET* Low *Tmin* Medium *Umax* Medium	*Yield* High	0.491	1.0	1.0

### Rules from Sudanian Zone

In the Sudanian Zone, the most favorable conditions for high tomato yield were either *ET* Low, *Tmin* medium, and *Umax* High or *Tmax* medium, *Umax* Medium, and *RR* High. Tomato yield in the Sudanian Zone was also high when *ET* was low, *Umax* Medium, and *RR* Medium, or *Tmax* Medium, *ET* low, and *RR* Medium. High yield was also achieved in this Zone when *Tmax*, *Tmin*, and *RR* were medium (Table 4).

More explicitly, the first rule dictated that tomato yield was high when *ET* was less than 3.009 mm, *Tmin* was between 19.024 and 25.120°C, and *Umax* was greater than 94.264%. The yield was also high when *Tmax* was within 31.733 and 37.793°C, and *Umax* between 52.850 and 94.264%, with *RR* greater than 0.323 mm. The third rule stated that high yield is obtained with *ET* less than 3.009 mm and *Umax* in the interval 52.850 and 94.264 mm and *RR* within ]0.109–0.323 mm[. According to the fourth rule, the yield was high with *Tmax* within ]31.733–37.793°C[, and *ET* less than 3.009 mm and *RR* within ]0.109–0.323 mm[. The last rule of the Zone suggested *Tmax* between 31.733 and 37.793°C, and *Tmin* between 19.024 and 25.120°C, and *RR* within ]0.109–0.323 mm[ for high tomato yield.

### Rules from Sudano–Guinean Zone

In the Sudano-Guinean Zone, only four rules met the stated conditions.

Tomato yield was high either when *ET* was Low, *Tmin* and *RR* Medium, or *ET* low, *Tmin* and *Umax* Medium, or *Tmax*, *Tmin* and *Umax* Medium, or *Tmin*, *Umax*, and *Sun* Medium. The first rule specified that the yield was above 322.760 Kg/ha in this area when *ET* is less than 2.387 mm, and *Tmin* in the range ]21.968–23.825°C[ with *RR* between 4.504 and 8.347 mm. The second rule projected a high yield when *ET* is less than 2.387 mm, and *Tmin* in the interval 21.968–23.825°C with *Umax* within ]88.161–95.710%[. About the third rule, the yield was high when *Tmax* is between ]31.079–36.545°C[ and *Tmin* within ]21.968–23.825°C[ with *Umax* in ]88.161–95.710%[. Regarding the fourth rule, the yield was high with *Tmin*, *Umax*, and *Sun* within 21.968–23.825°C[, ]88.161–95.710%[ and ]4.664–7.994 h[ respectively.

### Rules from Guinean Zone

In the Guinean Zone, the yield was high either when *ET* was low, *Tmin* and *Sun* Medium, or *Tmax*, *Tmin*, and *RR* medium, or *ET* low, *Umax* and *Sun* medium, or *Tmin*, *Umax* and *Sun* Medium or *ET* low, *Tmin* and *Umax* Medium. The first rule defined that when the *ET* is less than 1.971 mm, and *Tmin* between 24.424 and 26.213°C with *Sun* within 4.664 and 7.528 h the yield was high and therefore greater than 3618.280 Kg/ha. The second rule considered a high yield with *Tmax* between 29.214–32.282°C, *Tmin* between 24.424 and 26.213°C, and *RR* between 1.331 and 3.250 mm. From the third rule, the yield was high with *ET* below 1.971 mm, *Umax* between 88.161–95.710%, and *Sun* between ]4.664–7.528h[. The fourth rule stated that tomato yield was high with *Tmin*, *Umax*, and *Sun* in the intervals ]24.424–26.213°C[, ]88.161–95.710%[ and ]4.664–7.528 h[ respectively. The last rule assumed high yield when *ET* is below 1.971 mm, and *Tmin* ranges from 24.424 to 26.213°C and *Umax* between 88.161–95.710%.

## Discussion

The present study linking climatic variables to tomato yield is an original analysis of one of the most important vegetables in the Republic of Benin. The results indicated that conditions conducive to high tomato yield were average for most of the climatic parameters considered in this study. However, the rainfall was high in the Sudanian Zone. This is quite normal because this Zone’s monthly rainfall is lower than that in the other two Zones (Tables 1–3). Thus, the high rainfall threshold in the Sudanian Zone is below the average of the other Zones. The best yield patterns were observed in the Guinean Zone, followed by the Sudanian Zone. Performance in the Sudano-Guinean Zone was poor compared to the other two Zones. Thus, considering climatic data, the Sudano-Guinean Zone is unsuitable for tomato production. Our results align with those of Dwamena [27], who used multiple regression to assess the impact of minimum temperature, maximum temperature, and relative humidity variations on maize, cassava, and yam yields in Ghana. The results indicated that increased rainfall does not produce higher cassava yields [28]. Going in the same direction, Zhou and Guo [29, 30] demonstrated that high precipitation during flowering limits tomato growth. Indeed, heavy rainfall can lead to crops’ waterlogging, affecting crop roots’ respiration [29, 30]. Our results indicated that high tomato yield was associated with low *ET* in the three areas. *ET* refers to the loss of water through evaporation from the soil and transpiration by the plants themselves. High *ET* induces water stress for tomato plants, losing more water than they can absorb through their roots [31]. This leads to plant wilting, reduced growth, reduced fruit size, and eventually plant death if the water stress persists. When tomato plants suffer from water stress due to high *ET*, fruit production can be reduced. Flowers may abort before giving fruits, and growing fruits may dry out and fall prematurely. High *ET* affects the quality of tomato fruits [32]. Fruits become smaller, less juicy, and less flavorsome due to reduced water content. In addition, tomato plants subjected to prolonged water stress due to high *ET* become more vulnerable to disease. A weakened root system can be more susceptible to soil-borne infection, leading to diseases such as downy mildew or root rot. As a result, tomato plants subjected to high *ET* may not give high yields. We also found that the temperature and humidity were average in the regions with high yields. Our results are in line with the findings of several previous studies. Ezin [33] worked on food security under the threat of climate change in Benin. They experimented with three regions of the country (Cotonou, Bohicon, and Natitingou) and applied two treatments: 40°C considered as high and 27°C as normal. The study concluded that high temperature caused flower abortion and desiccation, resulting in low tomato yield [33]. Another study by Ayankojo [34] produced similar results. The study found that high temperatures resulted in lower fruit production and yield reduction. Similarly, Bhandari [35] observed that tomato production per hectare decreased when the maximum temperature was above 28°C. Tomatoes are sensitive to heat stress, which leads to reduced fruit production. However, Fernanda [36] found that temperature in Portugal is highly important in tomato yield prediction and negatively affects productivity from 21°C. They also observed that yield decreased with increasing relative humidity. According to Bhandari [35], yield increased when relative humidity was between 75% and 95%, which is consistent with our findings. In the same line, Fernanda [36] reported that humidity values greater than 71% positively impacted the average prediction of tomato yield. Furthermore, the results of this study are partially in line with those of Supro [16]. These authors worked on rice yield prediction and optimization using the Apriori algorithm and neural networks to improve agribusiness. They established several associations, including one which states that paddy rice yield was high in the Larkana region of India when humidity was medium. A high humidity environment is more likely conducive to the appearance of pests and diseases, resulting in reduced crop yields [29, 30]. However, our results could be more consistent with the findings of Rao [14], who established relationships between climatic and soil parameters and paddy yield in the Nanjangud taluk region of India with the ECLAT algorithm. They reported high yield when temperature and rainfall were high while soil pH and Nitrogen were medium. It can be justified by the fact that rice and tomato are different species with specific requirements. For example, in terms of water, rice is more water-consuming than tomatoes. The water requirement for tomatoes is between 1.62 and 4.58 mm per day [37]. However, according to Aryal [38], rice requires an average of 7.905mm of water daily. Therefore, while rice yields are high with high rainfall, tomato yields are high with low rainfall. In addition, the study areas are different, and the author did not give details on the different threshold values used to classify the attributes as low, medium, and high. This work is in the same direction as Ranjani [39], which used association rule algorithms to set up a farmer recommendation system. This system utilizes information about soil, weather, region, season, and past production to recommend the most profitable crops for cultivation in the appropriate environmental conditions. By presenting a comprehensive list of potential crops, the system aids farmers in deciding which crop to grow. Moreover, the system incorporates historical production data, allowing farmers to gain insight into the market demand and cost of different crops.

A variation in tomato yields was observed between different areas. Several factors may explain this variability. Changing climatic parameters significantly affect tomato yield. Areas benefiting from favorable climatic conditions, such as moderate temperatures, adequate rainfall, and optimal sunshine, tend to have higher yields than regions subject to extreme or unfavorable weather conditions. In addition, soil nutrient composition plays an important role in tomato production [40]. Furthermore, when tomato production is not carried out in managed environments, plants may be confronted with varying pest and disease infestation levels, which harms its yield. Pests such as aphids, nematodes, or diseases such as downy mildew, bacterial wilt, and fungal infections can lead to yield losses if not managed effectively [41]. In addition, the choice of tomato varieties or cultivars influences yield variations [42]. Different cultivars have varying genetic characteristics such as disease resistance, tolerance of environmental conditions, or productivity [42]. Thus, selecting appropriate cultivars adapted to specific regions helps optimize yields. Similarly, advanced agricultural technologies, such as greenhouse cultivation, precision irrigation, controlled environment systems, and improved post-harvest handling, can contribute to variations in tomato yields.

The present study has some limitations. Besides the climatic parameters that influence the yield, there is also the soil pH and the nutrient content in the soil. These different parameters have yet to be addressed at the moment. However, our research constitutes an essential departure point to implement the rules found in experiments with various fertilizers of different natures and doses to predict the yield of tomatoes based on all these parameters in real time.

## Conclusion

This paper used the Frequent Patterns Growth algorithm to establish rules between yield and climate parameters in Benin’s three agroecological Zones. The database comprised climatic and yield data collected over 26 years in Benin. The rules obtained revealed that the attributes giving high tomato yield were variable from one region to another. In particular, rainfall was high in the Sudanian Zone but low in the other two areas. On the other hand, the attributes minimum temperature, maximum temperature, and maximum humidity were medium regardless of the Zone considered. The best yield patterns were observed in the Guinean area. This work can be extended to other vegetables requiring approximately the same climatic conditions as tomatoes. This study can also be improved by taking data specific to each growth phase of the tomato in combination with fertilizer.
